# Inhibition of the JAK2/STAT3 Pathway Attenuates D‐Galactose‐Induced Nucleus Pulposus Cell Senescence and Intervertebral Disc Degeneration

**DOI:** 10.1155/sci/3373211

**Published:** 2025-12-09

**Authors:** Weidong Liang, Shuwen Zhang, Xiaoyu Cai, Yao Wang, Honggang Hao, Kup Ya, Jun Sheng, Weibin Sheng

**Affiliations:** ^1^ Department of Spine Surgery, The First Affiliated Hospital of Xinjiang Medical University, Urumqi, 830054, Xinjiang Uygur Autonomous Region, China, xjmu.edu.cn; ^2^ Department of Spine Surgery II, People’s Hospital of Xinjiang Uygur Autonomous Region, Urumqi, 830000, Xinjiang Uygur Autonomous Region, China, xjrmyy.com

**Keywords:** intervertebral disc degeneration, JAK, nucleus pulposus cell, senescence-related secretory phenotype, STAT3

## Abstract

**Objective:**

This study aimed to investigate the effect of Janus kinase 2 (JAK2)/signal transducer and activator of transcription 3 (STAT3) pathway inhibition on D‐galactose (D‐gal)‐induced senescence in nucleus pulposus cells (NPCs) and its potential to delay intervertebral disc degeneration (IVDD), as well as to investigate the underlying mechanisms.

**Methods:**

A cellular senescence model was established by treating rat NPCs with D‐gal. The model was then intervened with a JAK2/STAT3 pathway inhibitor (ruxolitinib) or JAK2‐specific small interfering RNA (siRNA). Cellular senescence was evaluated by senescence‐associated β‐galactosidase (SA‐β‐gal) staining. The expression of senescence markers (p16, p21, and p53), extracellular matrix (ECM) components (aggrecan and collagen II), catabolic enzymes (ADAMTS‐4, ADAMTS‐5, MMP‐3, and MMP‐13), and JAK2/STAT3 pathway proteins was analyzed by western blotting and immunofluorescence. The levels of inflammatory factors (interleukin [IL]‐1β, IL‐6, and tumor necrosis factor‐α [TNF‐α]) and advanced glycation end‐products (AGEs) were measured by enzyme‐linked immunosorbent assay (ELISA). Cell proliferation, apoptosis, and cell cycle distribution were assessed using cell counting kit‐8 (CCK‐8) and flow cytometry. In a parallel in vivo study, a rat model of IVDD was induced by D‐gal and treated with the JAK inhibitor. Disc degeneration was evaluated by magnetic resonance imaging (MRI) and histopathological examination after 8 weeks.

**Results:**

Both in vitro and in vivo, inhibition of the JAK2/STAT3 pathway, either pharmacologically or genetically, effectively attenuated D‐gal‐induced effects. It suppressed the phosphorylation of STAT3, reduced the expression of SA proteins (p16, p21, and p53), ECM catabolic enzymes (ADAMTS‐4, ADAMTS‐5, MMP‐3, and MMP‐13), and proinflammatory cytokines (IL‐1β and IL‐6). Consequently, this inhibition decreased SA‐β‐gal positivity, alleviated cell cycle arrest and apoptosis, and enhanced the synthesis of aggrecan and collagen II in NPCs. In the rat model, JAK inhibitor treatment improved MRI scores, restored disc signal intensity, and ameliorated histopathological degeneration.

**Conclusion:**

: Inhibition of the JAK2/STAT3 pathway reduced the expression of inflammatory factors and oxidative stress markers in D‐gal‐treated NPCs. It also suppressed ECM degradation and apoptosis, delayed cellular senescence, and attenuated the progression of IVDD in rats.

## 1. Introduction

Intervertebral disc degeneration (IVDD) is a prevalent condition that significantly affects the physiological and psychological well‐being of patients, while also imposing substantial economic burdens on families and society [1]. Within intervertebral discs, pathological changes such as reduced cell viability and function, extracellular matrix (ECM) metabolic imbalance, and chronic pain are closely linked to persistent inflammation [2]. Potassium channels have been identified as protective factors for disc cells under ischemic conditions and may also contribute to mechanotransduction [3]. In addition, growth factors, anticatabolic agents, and mesenchymal stem cells have been investigated for their potential in preventing and managing IVDD [4–6].

Senescent cells contribute to the chronic inflammatory environment of degenerative tissues by expressing and secreting components of the senescence‐associated secretory phenotype (SASP), which promote the recruitment of inflammatory cells. Modulating SASP may offer a strategy for anti‐aging interventions targeting degenerative diseases [7]. Nucleus pulposus cells (NPCs), which have limited proliferative capacity and a short lifespan, are particularly susceptible to senescence. Oxidative stress, mechanical overload, and metabolic disturbances can lead to persistent DNA damage and chromosomal alterations, thereby accelerating the senescence of these cells [8].

Reducing cellular senescence has the potential to diminish SASP expression. Novais et al. [8] demonstrated that cyclin‐dependent kinase inhibitor 2A (p16^INK4A^) plays a critical role in initiating and maintaining cellular senescence. Targeted silencing of p16^INK4A^ was shown to reduce senescence and suppress the expression of SASP‐related factors, including interleukin (IL)‐1β, IL‐6, C–C motif chemokine ligand 2, and transforming growth factor‐β1. This may help preserve the balance between anabolic and catabolic processes in the ECM of the intervertebral disc.

The signal transducer and activator of transcription (STAT) pathway, a key intracellular signaling mechanism for various cytokines, plays an important role in regulating inflammatory responses. Phosphorylation of STAT is mediated by upstream Janus kinases (JAKs), which are activated upon cytokine binding and receptor dimerization [9]. This JAK‐STAT signaling axis facilitates intracellular communication of cytokine signals, including those related to SASP, potentially disrupting paracrine signaling networks. Although this pathway is recognized for its involvement in inflammation, the specific role of SASP in IVDD remains insufficiently characterized. Furthermore, the contribution of NPCs to disc degeneration via SASP‐related mechanisms has not been extensively investigated.

This study aimed to evaluate whether inhibition of the JAK2/STAT3 signaling pathway could suppress SASP expression, attenuate NPC senescence, and delay the progression of IVDD, while also exploring the associated molecular mechanisms.

## 2. Materials and Methods

### 2.1. Equipment and Reagents

Ruxolitinib (Macklin, R849099), fetal bovine serum (FBS; Excell, FND500), Type II collagenase (Sigma, C2‐28‐100MG), cell counting kit‐8 (CCK‐8; TransGen, FC101‐03), Dulbecco’s modified eagle medium (DMEM)/F12 medium (Hyclone, SH30023.01) were used in this study. Primary antibodies included collagen Type II (Abcam, ab34712) and aggrecan (Thermo, MA3‐16888). Additional reagents included phenylmethylsulfonyl fluoride (PMSF; Boster, AR1178) and enzyme‐linked immunosorbent assay (ELISA) kits for IL‐1β (Multisciences, EK301B/4‐96), IL‐6 (Multisciences, EK306/3‐96), tumor necrosis factor‐α (TNF‐α; Multisciences, EK382/3‐96), matrix metalloproteinase‐3 (MMP‐3; Cusabio, CSB‐E07410r), and MMP‐13 (Cusabio, CSB‐E07412r).

Other materials and instruments included molecular weight marker (Solarbio, PR1910), a microplate reader (Bio‐Rad, xMark), RFect small interfering RNA (siRNA)/miRNA transfection reagent (Biog, 11012), BeyoClick EdU‐488 kit (Beyotime, C0071S), a high‐speed low‐temperature centrifuge (Thermo Fisher Scientific), an enzyme labeling instrument (Bio‐Rad, USA), a laser confocal microscope (Carl Zeiss, Germany), a flow cytometer (Guilin Unite Medical Electronics Co., Ltd.), a protein transfer apparatus (Bio‐Rad, USA), an electrophoresis instrument (Beijing Liyuyi Biotechnology Co., Ltd.), a decolorizing shaker (Chirin Bell Instrument Co., Ltd.), a magnetic resonance imaging (MRI) instrument, and antibody information (Table 1).

**Table 1:** Antibody information.

Antibody	Supplier	Catalog number	KD	Dilution rate
Goat anti‐rabbit IgG	Abcam	ab205718		1:5000
Goat anti‐mouse IgG	Abcam	ab205719		1:10,000
Beta‐actin	Sino	100166‐MM10	42	1:1000
p16INK4A	Abcam	ab189034	16	1:800
ADAMTS4	Affinity	DF6986	90	1:400
ADAMTS5	Affinity	DF13268	60	1:400
p21	Proteintech	28248‐1‐AP	18	1:400
Phospho‐p53	Affinity	AF3075	44	1:400
p53	Affinity	AF0879	44	1:500
JAK2	Affinity	AF6022	130	1:500
Phospho‐JAK2	Affinity	AF3022	131	1:400
STAT3	Abcam	ab68153	80	1:1000
Phospho‐STAT3	Abcam	ab32143	92	1:800
COX‐2	Proteintech	66351‐1‐Ig	65	1:400

### 2.2. Isolation and Culture of NPCs

Male Sprague–Dawley rats (12 weeks old; 230 ± 20 g) were obtained from the Experimental Animal Center of Xinjiang Medical University (Xinjiang, China). NPCs were isolated from the coccygeal intervertebral discs of three euthanized rats. Nucleus pulposus (NP) tissue fragments were digested with 0.1% Type II collagenase at 37°C for 25 min, followed by centrifugation at 1000 rpm for 5 min. The resulting cell pellet was resuspended in DMEM/F12 supplemented with 10% FBS and 1% penicillin‐streptomycin s olution (Thermo Fisher Scientific). Cells were cultured in a humidified incubator at 37°C with 5% CO_2_ and subcultured upon reaching 80%–90% confluence.

NPCs were characterized by immunofluorescence detection of Type II collagen expression during the culture process. The immunofluorescence results confirmed abundant cytoplasmic expression of Type II collagen in NPCs (Figure 1).

Figure 1Immunofluorescence analysis of Type II collagen expression in NPCs. (a) Representative images of Type II collagen immunofluorescence staining in each experimental group. (b) Quantitative analysis of collagen II expression levels across groups. Data are presented as mean ± standard deviation.  ^∗^ indicates statistically significant differences compared with the control group (*p* < 0.05); # indicates statistically significant differences between the two indicated groups (*p* < 0.05).

**Figure:**
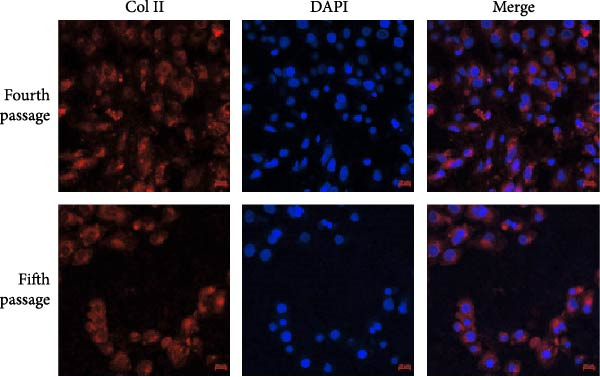
(a)

**Figure:**
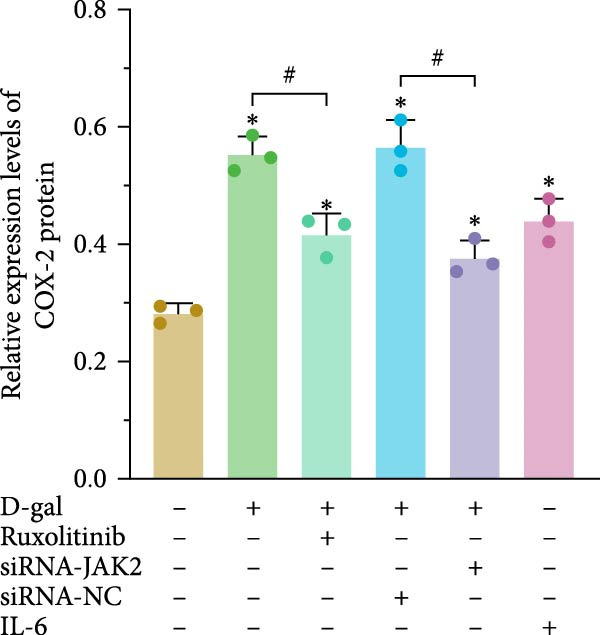
(b)

### 2.3. Screening of Intervention Conditions

#### 2.3.1. siRNA Transfection Conditions

NPCs (5 × 10^4^ cells/mL) were plated and transfected with a complex of siRNA and RFect transfection reagent at a final siRNA concentration of 0.01 μM. Following transfection, fluorescence microscopy was employed to observe cellular uptake and identify optimal transfection conditions.

#### 2.3.2. Screening for Optimal siRNA Sequence

NPCs were seeded into six‐well plates and maintained in a standard cell culture incubator. Cells were transfected with either negative control siRNA or JAK2‐targeting siRNA sequences directed at three different regions, using the previously determined optimal transfection parameters. A control group without siRNA was also included. Each experimental group was performed in triplicate. After treatment, 1 mL of TRIzol reagent was added to each well to lyse the cells, ensuring uniform coverage. Culture flasks were gently agitated until complete cell lysis was achieved, and the lysate was collected in 1.5 mL microcentrifuge tubes. Total RNA was extracted, and the expression of JAK2 was quantified using quantitative reverse transcription polymerase chain reaction (qRT‐PCR). Based on the expression profiles, the siRNA sequence JAK2‐siRNA‐3389 was selected for subsequent experiments. JAK2 expression levels for each group are presented in Table 2.

**Table 2:** JAK2 expression levels in each group ($\overset{-}{x}$ ± *s*, *n* = 3).

Group	JAK2 expression level
Control	1.001 ± 0.057
siRNA‐NC	1.370 ± 0.174^a^
JAK2‐siRNA‐1654	0.184 ± 0.019^ab^
JAK2‐siRNA‐1848	0.211 ± 0.037^ab^
JAK2‐siRNA‐3389	0.080 ± 0.012^ab^

Abbreviations: JAK, Janus kinase; siRNA‐NC, small interfering RNA‐negative control.

^a^Compared with the control group, p < 0.05.

^b^Compared with the siRNA‐NC group, p < 0.05.

#### 2.3.3. Time‐Point Screening for Interventions

NPCs (5 × 10^4^ cells/mL) were seeded into 24‐well plates and allowed to adhere overnight. Senescence was induced by treating cells with 10 mg/mL D‐(+)‐galactose or 10 ng/mL IL‐6 for 24, 48, and 72 h. A control group was cultured under identical conditions without any treatment. Cellular senescence in all groups was assessed using SA β‐galactosidase (SA‐β‐gal) staining.

### 2.4. Experimental Grouping

#### 2.4.1. Cell Model

Third‐generation NPCs were retrieved and grouped based on different interventions as follows:

1. Control group: no intervention.
2. D‐galactose (D‐gal) senescence model group: treated with 10 mg/mL D‐gal.
3. D‐gal + JAK inhibitor group (JAK I): treated with 10 mg/mL D‐gal and 1 μM ruxolitinib.
4. D‐gal + siRNA‐negative control (siRNA‐NC) group: transfected with 0.1 μM siRNA‐NC.
5. D‐gal + JAK2‐siRNA group: transfected with 0.1 μM JAK2‐targeting siRNA.
6. IL‐6 group: treated with 10 ng/mL IL‐6.

Groups 2–6 received the respective interventions during the passaging process from the third to the fourth generation and were continuously cultured until the sixth generation. At the end of the culture period, sixth‐generation cells were collected for analysis of relevant markers.

#### 2.4.2. Animal Model

Adult male Sprague–Dawley rats (200–250 g) were obtained from the Animal Experiment Center of Xinjiang Medical University and randomly assigned to the following groups:

1. Control group: received daily subcutaneous and intraperitoneal injections of equal volumes of saline for 8 weeks.
2. D‐gal degeneration model group: received daily subcutaneous injections of D‐gal (200 mg/kg) and intraperitoneal injections of equal volumes of saline for 8 weeks.
3. D‐gal + JAK inhibitor group (JAK I): received daily subcutaneous injections of D‐gal (200 mg/kg) and intraperitoneal injections of ruxolitinib (15 mg/kg), administered twice daily for 8 weeks.

After 8 weeks of treatment, all animals were euthanized with an overdose of 0.1% sodium pentobarbital, and caudal intervertebral discs were harvested for specimen analysis.

### 2.5. CCK‐8 Detection of Cell Proliferation

Rat NPCs were isolated and seeded into 96‐well plates. After overnight incubation to allow cell attachment, group‐specific interventions were applied, with five replicates per group. After 72 h of treatment, CCK‐8 reagent was added to each well, and the plates were incubated at 37°C for 1 h. Optical density (OD) values at 450 nm were measured using a microplate reader.

### 2.6. Flow Cytometry Assay for Apoptosis Detection

Apoptosis in treated NPCs was assessed using double staining with the Annexin V‐PE/7‐AAD apoptosis detection kit (BD Biosciences, 559763). Cells were harvested, washed three times with cold phosphate‐buffered saline (PBS), and resuspended in 100 μL of 1 × binding buffer at a concentration of 1 × 10^5^ cells. Subsequently, 5 μL of Annexin V‐PE and 5 μL of 7‐AAD were added to the suspension, and the cells were incubated at room temperature in the dark for 15 min. After staining, 400 μL of 1 × binding buffer was added, and samples were analyzed using a BD FACSVerse flow cytometer.

### 2.7. Flow Cytometry Cell Cycle Assay

Treated NPCs were collected, washed with cold PBS, and resuspended in 500 μL of cold PBS. Fixation was performed by adding 3.5 mL of cold 80% ethanol, followed by overnight incubation at 4°C. After fixation, cells were washed twice with cold PBS and resuspended in 500 μL of PI/RNase staining buffer (BD, 550825). The suspension was incubated in the dark at 4°C for 30 min. Red fluorescence was measured by flow cytometry using the BD FACSVerse instrument with an excitation wavelength of 488 nm. Cellular DNA content and light scattering data were analyzed using ModFit 5.0 software.

### 2.8. Detection of Cellular Senescence by β‐gal Staining

NPCs (5 × 10^4^ cells/mL) were seeded into 24‐well plates and incubated overnight to allow adherence. Cells were then assigned to their respective intervention groups. Following treatment, 500 μL of β‐gal fixative was added to each well, and the cells were fixed at room temperature for 15 min. The fixative was aspirated, and cells were washed three times with PBS. Subsequently, 0.5 mL of staining solution was added to each well, and the plates were incubated at 37°C overnight. Under microscopy, senescent cells appeared green, while nonsenescent cells remained unstained. The percentage of senescent cells was calculated as: (number of senescent cells per unit field of view/total number of cells) × 100.

### 2.9. Immunofluorescence Detection of Aggrecan and Type Collagen II

NPCs were collected, assigned to intervention groups, and applied dropwise onto adhesive slides, with three replicates per group. Cells were washed once with PBS, fixed with 4% paraformaldehyde for 10 min, and washed three times with PBS. Permeabilization was performed using 0.5% Triton X‐100 for 20 min, followed by three additional PBS washes.

At room temperature, cells were blocked with 1% bovine serum albumin for 40 min. After removal of the blocking solution, primary antibodies against aggrecan and Type II collagen were applied dropwise and incubated overnight at 4°C. Slides were then washed three times with PBS.

Secondary antibodies Alexa Fluor 594‐conjugated goat anti‐rabbit IgG H&L and Alexa Fluor 488‐conjugated goat anti‐mouse IgG H&L, were applied dropwise and incubated at 37°C for 1 h, followed by three washes with PBS.

4,6‐Diamidino‐2‐phenylindole was added for nuclear staining and incubated at room temperature in the dark for 5 min. After washing three times with PBS in the dark, coverslips were mounted using 50% glycerol. The expression of aggrecan and Type II collagen was visualized using a laser confocal microscope.

### 2.10. ELISA for Detection of SASPs

After allowing the plate to equilibrate at room temperature for 30 min, 100 μL of standard solution was added to six designated wells. Subsequently, 100 μL of serum samples was added to the remaining wells, followed by 50 μL of enzyme labeling solution. The plate was incubated at room temperature for 90 min and then washed five times.

Following the washes, 50 μL each of Solution A and Solution B was added to each well, and the plate was incubated in the dark for 15 min. The reaction was terminated by adding 50 μL of stop solution to each well. OD values at 450 nm were measured using a microplate reader.

The analytes assessed included IL‐1β, IL‐6, TNF‐α, MMP‐3, and MMP‐13.

### 2.11. Protein Immunoblotting

Total cellular proteins were extracted, and concentrations were determined using a bicinchoninic acid protein assay kit. Equal amounts of protein were separated by sodium dodecyl sulfate‐polyacrylamide gel electrophoresis and transferred onto polyvinylidene fluoride membranes. Membranes were blocked in a buffer containing 5% skim milk for 2 h at room temperature.

Following blocking, membranes were washed with PBS and incubated overnight at 4°C with the appropriate primary antibodies. After incubation, membranes were washed again with PBS and incubated with horseradish peroxidase‐labeled goat anti‐rabbit secondary antibodies for 2 h at room temperature.

Protein bands were developed using an enhanced chemiluminescence reagent and visualized using an imaging system.

### 2.12. MRI Imaging

At Weeks 4 and 8, rats in each group were weighed and anesthetized. MRI of the caudal vertebrae was performed using a 1.5 T system with the following parameters: repetition time of 3000 ms, echo time of 80 ms, field of view of 200 mm, and slice thickness of 1.4 mm. T2‐weighted images of the intervertebral discs were acquired and archived using dedicated imaging software.

Following image acquisition, intervertebral discs were graded according to the Pfirrmann classification system to evaluate disc degeneration. Statistical analysis was performed on the grading data to assess significance.

### 2.13. Histopathological Testing

Histopathological scoring was performed based on hematoxylin and eosin (HE) staining and Senna solid green staining. Caudal disc specimens were fixed in 4% paraformaldehyde for 24 h, dehydrated through a graded ethanol series, cleared with xylene, embedded in paraffin, and sectioned at a thickness of 5 μm.

Paraffin sections were rehydrated and stained with hematoxylin for 5 min. After staining, sections were differentiated with 1% hydrochloric acid in ethanol for 20 s and then blued in 1% ammonia solution. Counterstaining was carried out using 1% eosin for 5 min. The sections were then dehydrated through an ascending ethanol series and mounted with neutral gum.

Prepared slides were examined under a BH‐2 optical microscope.

### 2.14. Immunohistochemical Staining

Paraffin‐embedded tissue sections were deparaffinized, rehydrated, and incubated in freshly prepared 3% hydrogen peroxide (H_2_O_2_) for 10 min at room temperature to inhibit endogenous peroxidase activity. Antigen retrieval was performed using microwave heating for 20 min. Sections were then blocked with serum from nonimmunized animals at 37°C for 15 min.

Primary antibodies were applied and incubated overnight at 4°C. After washing with PBS, biotinylated secondary antibodies were added dropwise and incubated at 37°C for 40 min. Color development was achieved using freshly prepared 3,3^′^‐diaminobenzidine chromogen solution, and the reaction was terminated under microscopic observation. Counterstaining was performed using hematoxylin, followed by rinsing with distilled water.

Sections were dehydrated through a graded ethanol series, cleared in xylene for 5 min, and sealed with a neutral adhesive. Images were captured from five randomly selected fields under a light microscope.

### 2.15. Statistical Analysis

Statistical analyses were performed using IBM SPSS Statistics version 19.0 (IBM Corp., Armonk, NY, USA), and data visualization was carried out using GraphPad Prism version 5.0 (GraphPad Software Inc., La Jolla, CA, USA). Data are presented as mean ± standard deviation from three independent experiments. One‐way analysis of variance (ANOVA) was used for comparisons among groups with normally distributed data. For nonnormally distributed data, the nonparametric rank‐sum test was applied. A *p*‐value of less than 0.05 was considered statistically significant.

## 3. Results

### 3.1. Role of the JAK2/STAT3 Pathway in D‐gal Induced Senescence of NPCs

#### 3.1.1. Quantification of AGE in Different NPC Groups

The expression of advanced glycation end‐products (AGEs) was quantified across experimental groups to evaluate their involvement in NPC senescence. The measured AGE levels in the control, D‐gal, JAK inhibitor, siRNA‐NC, D‐gal + JAK2‐siRNA, and IL‐6 groups were 0.286 ±  0.049, 1.153 ± 0.028, 0.534 ± 0.077, 1.109 ± 0.035, 0.390 ± 0.087, and 0.783 ± 0.075, respectively.

A significant increase in AGE expression was observed in the D‐gal, siRNA‐NC, and IL‐6 groups compared to the control group (*p* = 0.001 for all). Treatment with a JAK inhibitor significantly reduced AGE expression in the D‐gal + JAK inhibitor group compared to the D‐gal group (*p* = 0.001). Similarly, JAK2 silencing significantly decreased AGE expression in the D‐gal + JAK2‐siRNA group compared to the D‐gal + siRNA‐NC group (*p* = 0.001).

#### 3.1.2. JAK Inhibitor Intervention or Targeted Silencing of JAK2 Delays D‐gal‐Induced Senescence of NPCs

β‐gal staining was conducted in each group, with positive staining considered indicative of cellular senescence (Figure 2a). The D‐gal group exhibited a significantly increased proportion of β‐gal‐positive cells (63.3% ± 5.0%) compared to the control group (4.9% ± 2.3%, *p* < 0.001). A similar increase was noted in the siRNA‐NC group.

Figure 2JAK inhibitor intervention or targeted silencing of JAK2 delays D‐gal‐induced senescence of NPCs. (a) Statistical analysis of the positive rate of β‐galactosidase staining for cellular senescence. (b) Relative expression levels of aging‐related proteins. For all variables, normality was assessed using the Kolmogorov–Smirnov (K–S) test. One‐way ANOVA was conducted to compare group means, followed by LSD or Tamhane’s post hoc tests where appropriate.  ^∗^ indicates a statistically significant difference compared with the control group (*p* < 0.05); # indicates a statistically significant difference between the indicated groups (*p* < 0.05). *n* = 3.

**Figure:**
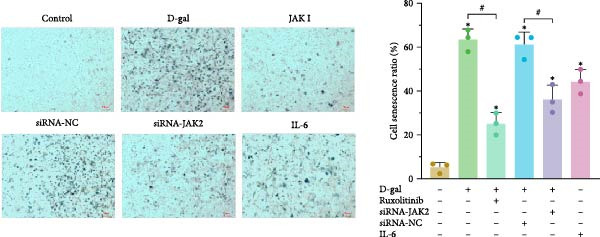
(a)

**Figure:**
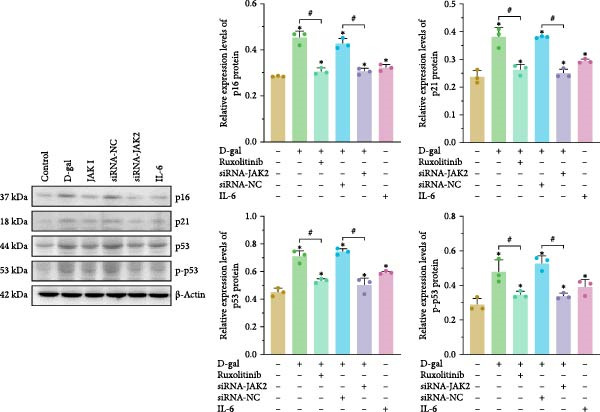
(b)

Following treatment with the JAK inhibitor, the proportion of β‐gal‐positive cells in the D‐gal + JAK inhibitor group was significantly reduced compared to the D‐gal group (25.0% ± 5.1% vs. 60.9%  ± 5.8%, *p* < 0.001). In addition, JAK2 silencing significantly reduced the β‐gal‐positive rate in the D‐gal + JAK2‐siRNA group compared to the D‐gal + siRNA‐NC group (36.0% ± 6.4% vs. 60.9%  ±  5.8%, *p* < 0.001).

The expression of senescence‐related proteins, including p16, p21, p53, and phosphorylated p53 (p‐p53), was examined across groups (Figure 2b). Induction by D‐gal led to upregulation of these proteins, which was subsequently reduced following treatment with the JAK inhibitor or JAK2‐targeted siRNA. These findings indicate that D‐gal induces senescence in NPCs.

#### 3.1.3. Targeting JAK Reverses the Effects of D‐gal on NPC Proliferative, Apoptosis, and Cell Cycle Arrest

Cell viability was assessed in the control, D‐gal senescence, JAK I, siRNA‐NC, and D‐gal + JAK2‐siRNA groups using the CCK‐8 assay. A significant reduction in proliferation was observed in the D‐gal group compared with the control group (70.5 ± 4.7 vs. 100.0 ± 4.3, *p* < 0.001; Figure 3a). Treatment with the JAK inhibitor significantly increased cell viability compared to the D‐gal group (90.4 ± 5.9 vs. 70.5 ± 4.7, *p* < 0.001). In addition, JAK2 silencing led to increased proliferation in the D‐gal + JAK2‐siRNA group relative to the D‐gal + siRNA‐NC group (81.9 ± 4.4 vs. 67.5 ± 5.6, *p* < 0.001; Figure 3a).

Figure 3Targeting JAK reverses the effects of D‐galactose on NPC proliferation, apoptosis, and cell cycle arrest. (a) Cell viability was assessed using the CCK‐8 assay across treatment groups. (b) Apoptosis rates were evaluated by flow cytometry. (c) Cell cycle distribution was analyzed via flow cytometry. Data normality was assessed using the Kolmogorov–Smirnov (K–S) test. Group differences were evaluated by one‐way ANOVA, with post hoc comparisons performed using LSD or Dunnett’s T3 test, as appropriate.  ^∗^ indicates statistically significant differences between the two indicated groups (*p* < 0.05); *n* = 5.

**Figure:**
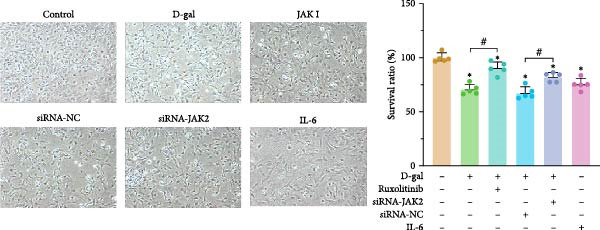
(a)

**Figure:**
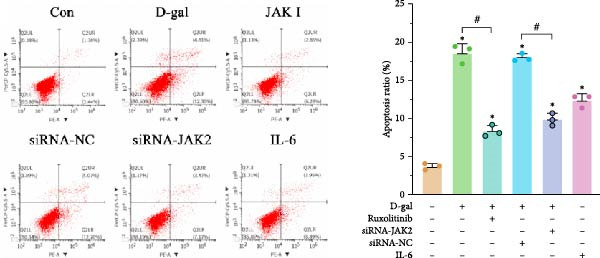
(b)

**Figure:**
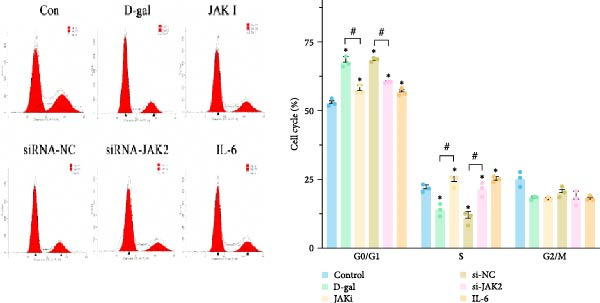
(c)

Apoptosis was evaluated by flow cytometry. A significant increase in apoptosis was detected in the D‐gal group compared to the control group (18.5% ± 1.2% vs. 3.7% ± 0.4%, *p* < 0.001; Figure 3b). Following JAK inhibitor treatment, the apoptosis rate was markedly reduced (8.3% ± 0.7% vs. 18.5% ± 1.2%, *p* < 0.001). Similarly, JAK2 silencing resulted in a substantial decrease in apoptosis in the D‐gal + JAK2‐siRNA group compared to the D‐gal + siRNA‐NC group (9.9% ± 0.8% vs. 18.0% ± 0.5%, *p* < 0.001; Figure 3b).

Cell cycle distribution analysis revealed a higher proportion of G_0_/G_1_‐phase cells in the D‐gal group than in the control group (67.8% ± 1.8% vs. 52.9% ± 1.4%, *p* < 0.001; Figure 3c), indicating cell cycle arrest. Treatment with the JAK inhibitor significantly decreased the G_0_/G_1_ population (57.5% ± 1.7% vs. 67.8% ± 1.8%, *p* < 0.001), consistent with enhanced DNA replication. JAK2 silencing also reduced the proportion of G_0_/G_1_‐phase cells compared to the siRNA‐NC group (60.3% ± 0.3% vs. 68.5% ± 0.7%, *p* < 0.001; Figure 3c).

### 3.2. JAK Inhibitor Intervention or Targeted Silencing of JAK2 Reduces the Expression of Inflammatory Factors in NPCs After D‐gal Induction

The upregulation of multiple proinflammatory cytokines contributes to the pathophysiological changes associated with IVDD. To determine whether JAK inhibition could suppress cytokine expression, the levels of IL‐1β, IL‐6, TNF‐α, and COX‐2 were measured.

In the D‐gal group, expression levels of IL‐1β, IL‐6, TNF‐α, and COX‐2 were significantly elevated compared to the control group (IL‐1β: 57.00 ± 5.47 vs. 30.61 ± 6.11; IL‐6: 29.53 ± 4.97 vs. 2.31 ± 1.69; TNF‐α: 11.17 ± 1.03 vs. 6.62 ± 1.11; COX‐2: 0.55 ± 0.03 vs. 0.28 ± 0.02; *p* < 0.001 for all comparisons).

Treatment with a JAK inhibitor significantly reduced the expression of IL‐1β and IL‐6 compared to the D‐gal group (IL‐1β: 36.78 ± 3.07 vs. 57.00 ± 5.47, *p* = 0.001; IL‐6: 9.76 ± 2.53 vs., 29.53 ± 4.97, *p* < 0.001; Supporting Information 1: Figure S1).

In the D‐gal + JAK2‐siRNA group, expression of IL‐1β, IL‐6, and COX‐2 was also significantly reduced compared to the D‐gal + siRNA‐NC group (IL‐1β: 39.37 ± 3.64 vs. 56.59 ± 0.35, *p* = 0.002; IL‐6: 15.73 ± 2.94 vs. 27.52 ± 1.42, *p* = 0.001; COX‐2: 0.38 ± 0.03 vs. 0.57 ± 0.05, *p* < 0.001).

However, the inhibitory effect on TNF‐α expression was less pronounced. In the JAK inhibitor group, TNF‐α levels were 9.44 ± 0.58 compared to 11.17 ± 1.03 in the D‐gal group (*p* = 0.081). Similarly, in the JAK2‐siRNA group, TNF‐α levels were 9.09 ± 0.33 compared to 10.68 ± 1.98 in the siRNA‐NC group (*p* = 0.103; Supporting Information 1: Figure S1).

### 3.3. JAK Inhibitor Intervention or Targeted Silencing of JAK2 Regulates the Metabolic Balance of the Extracellular Matrix

Immunofluorescence and ELISA analyses demonstrated that D‐gal significantly inhibited ECM anabolism and promoted catabolism. Specifically, D‐gal treatment led to a marked reduction in the expression of aggrecan and Col II compared to the control group (aggrecan: 32.22 ± 2.70 vs. 61.89 ± 1.26; Col II: 17.78 ± 3.47 vs. 39.61 ± 2.43; *p* < 0.001 for both; Figure 4).

Figure 4AK inhibitor intervention or targeted silencing of JAK2 can regulate the metabolic balance of the ECM. (a) Relative expressions of COX‐2, ADAMTS4, and ADAMTS5 were detected by western blot analysis. (b) The expressions of proteoglycan and Type II collagen were evaluated by immunofluorescence. For variables, K–S tests were first conducted. One‐way ANOVA was used to evaluate group effects, followed by LSD and Tamhane’s test for post hoc comparisons.  ^∗^ denotes a statistically significant difference compared with the control group (*p* < 0.05); # denotes a statistically significant difference between the two groups (*p* < 0.05); *n* = 3.

**Figure:**
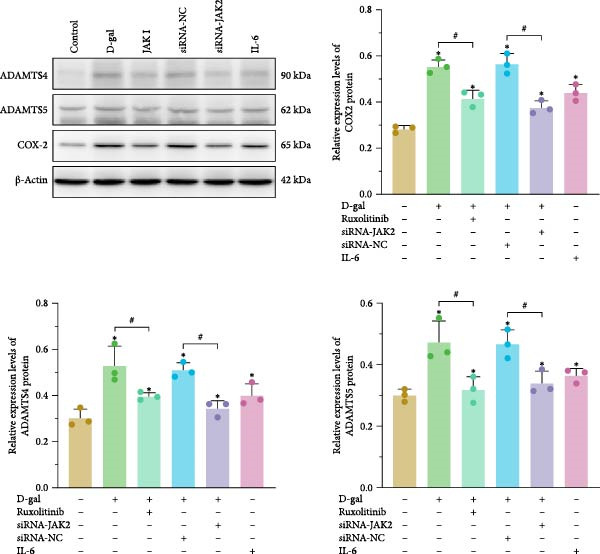
(a)

**Figure:**
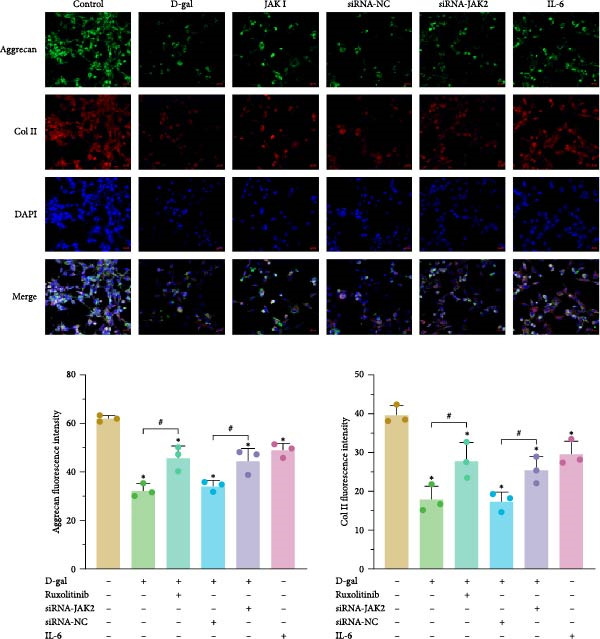
(b)

With increasing senescence of NPCs, there was a notable elevation in the secretion of MMP‐3, MMP‐13, and proteoglycan‐degrading enzymes ADAMTS4 and ADAMTS5 (MMP‐3: 0.971 ± 0.023 vs. 0.368 ± 0.059; MMP‐13: 425.73 ± 9.08 vs. 266.08 ± 18.26; ADAMTS4: 0.531 ± 0.083 vs. 0.473 ± 0.068; ADAMTS5: 0.303 ± 0.039 vs. 0.300 ± 0.020; *p* < 0.001, *p* < 0.001, *p* = 0.001, and *p* = 0.003, respectively). These results suggest that D‐gal induces ECM metabolic imbalance.

Compared to the control group, the expression levels of ADAMTS4 and ADAMTS5 were significantly reduced in the JAK inhibitor treatment group (ADAMTS4: 0.397 ± 0.016 vs. 0.531 ± 0.083, *p* = 0.005; ADAMTS5: 0.319 ± 0.042 vs. 0.554 ± 0.029, *p* = 0.001).

Similarly, in the D‐gal + JAK2‐siRNA group, the expression of both enzymes was lower compared to the D‐gal + siRNA‐NC group (ADAMTS4: 0.345 ± 0.034 vs. 0.511 ± 0.033, *p* = 0.001; ADAMTS5: 0.339 ± 0.039 vs. 0.467 ± 0.046, *p* = 0.003; Figure 4).

### 3.4. Reducing STAT3 Phosphorylation by Inhibiting JAK2 Expression Delays D‐gal‐Induced NPC Senescence

The interactions among ruxolitinib, cellular senescence, and STAT3 activation were examined by assessing phosphorylation within the JAK2/STAT3 pathway. Protein immunoblotting demonstrated that ruxolitinib significantly downregulated JAK2, phosphorylated JAK2 (p‐JAK2), and phosphorylated STAT3 (p‐STAT3) compared to the control group (JAK2: 0.437 ± 0.036 vs. 0.632 ± 0.007, *p* < 0.001; p‐JAK2: 0.461 ± 0.009 vs. 0.603 ± 0.023, *p* < 0.001; p‐STAT3: 0.451 ± 0.056 vs. 0.580 ± 0.017, *p* = 0.013; Figure 5).

Figure 5Reducing STAT3 phosphorylation by inhibiting JAK2 expression can delay D‐gal‐induced NPC senescence. (a) Immunoblotting bands of JAK2/STAT3 signaling pathway‐related proteins. (b) Relative expression levels of JAK2/STAT3 pathway‐related proteins. The normality of phosphorylated protein data was assessed using K–S tests. One‐way ANOVA was used to evaluate intergroup differences, followed by LSD and Tamhane’s tests for post hoc comparisons.  ^∗^ denotes a statistically significant difference compared with the control group (*p* < 0.05); # denotes a statistically significant difference between the two groups (*p* < 0.05); *n* = 3.

**Figure:**
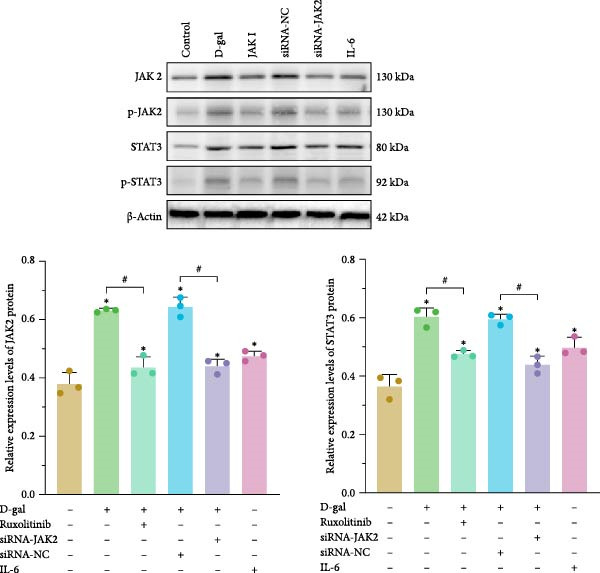
(a)

**Figure:**
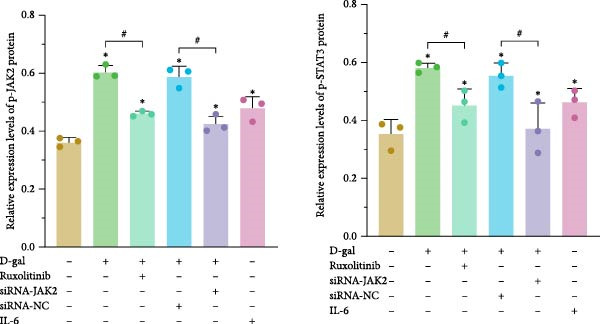
(b)

Similarly, JAK2 silencing significantly reduced p‐STAT3 expression compared to the D‐gal group (0.474 ± 0.013 vs. 0.603 ± 0.031, *p* < 0.001; Figure 5).

To further evaluate these interactions, normal NPCs were treated with IL‐6. IL‐6 stimulation led to increased levels of AGEs, a higher percentage of β‐gal‐positive senescent cells (44.233 ± 5.500 vs. 4.933 ± 2.294, *p* < 0.05), and elevated expression of SA genes including *p16*, *p53*, and *p21* (0.783 ± 0.075; 0.323 ± 0.013; 0.592 ± 0.011; 0.292 ± 0.007, respectively).

Cell cycle analysis revealed an increase in G_0_/G_1_‐phase arrest (56.763 ± 1.334). Furthermore, the expression of ECM catabolic enzymes and inflammatory mediators was significantly upregulated. These included ADAMTS4 (0.402 ± 0.049), ADAMTS5 (0.365 ± 0.022), MMP‐3 (0.449 ± 0.105), MMP‐13 (450.6 ± 53.6), IL‐1β (54.669 ± 9.420), IL‐6 (18.482 ± 3.475), TNF‐α (18.482 ± 3.475), and COX‐2 (0.440 ± 0.037).

These findings support that aberrant activation of the JAK2/STAT3 pathway contributes to the senescence of NPCs.

### 3.5. JAK Inhibitors Ameliorate Needled Disc Degeneration in Rats by Inhibiting the JAK2/STAT3 Pathway

#### 3.5.1. MRI Detection of D‐gal‐Induced Disc Degeneration in Rats

After 4 weeks of intervention, MRI revealed evidence of D‐gal‐induced disc degeneration in rats, characterized by reduced and heterogeneous T2 signal intensity within the NP (Supporting Information 2: Figure S2). By the eighth week, a significant increase in Pfirrmann grading scores was observed, confirming the successful establishment of a disc degeneration model (Supporting Information 2: Figure S2).

Following 8 weeks of JAK inhibitor treatment, MRI findings indicated partial restoration of NP signal intensity, suggesting that the JAK inhibitor exerted a protective effect against D‐gal‐induced IVDD in rats (Supporting Information 2: Figure S2).

#### 3.5.2. β‐gal Staining

**Figure 6:**
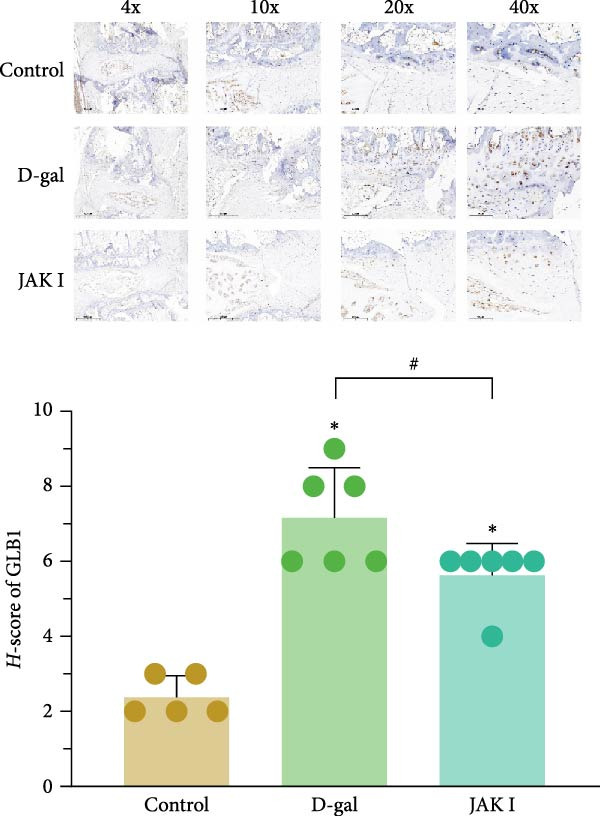
β‐Galactosidase staining. The normality of data was assessed using the K–S test. One‐way ANOVA was used to evaluate group differences, followed by LSD and Dunnett’s T3 tests for post hoc comparisons.  ^∗^ denotes a statistically significant difference compared with the control group (*p* < 0.05); # denotes a statistically significant difference between the two groups (*p* < 0.05).

The results indicated that GLB1 protein was predominantly expressed in the ECM of articular cartilage. In the control group, GLB1 protein showed limited nuclear staining, with lighter coloration and lower expression intensity (Figure 6). The D‐gal group exhibited a wider distribution of positive staining, deeper coloration, and higher GLB1 expression intensity (*p* < 0.001; Figure 6). The JAK inhibitor group showed lighter staining and a slightly lower intensity of positive expression relative to the D‐gal group (*p* = 0.019). Tissue sections were observed under a light microscope, and protein expression was scored accordingly.

The expression levels of aging‐related proteins were also analyzed. D‐gal induction upregulated the expression of p16, p21, p53, and p‐p53 in rat medullary tissues (0.57 ± 0.04, 0.44 ± 0.03, 0.76 ± 0.04, 0.85 ± 0.11 vs. 0.27 ± 0.02, 0.28 ± 0.03, 0.54 ± 0.04, 0.62 ± 0.09; *p* < 0.001 for all comparisons). Treatment with the JAK inhibitor reduced the expression of these proteins (0.47 ± 0.04, 0.35 ± 0.03, 0.65 ± 0.01, 0.72 ± 0.18 vs. 0.57 ± 0.04, 0.44 ± 0.03, 0.76 ± 0.04, 0.85 ± 0.11; *p* < 0.001, *p* < 0.001, *p* < 0.001, and *p* = 0.020, respectively; Supporting Information 3: Figure S3).

#### 3.5.3. JAK Inhibitors Suppress D‐gal‐Induced Expression of Inflammatory Factors and Oxidative Stress In Vivo

ELISA revealed that D‐gal administration significantly upregulated the expression of AGEs, IL‐1β, IL‐6, TNF‐α, and COX‐2 in rat intervertebral discs (AGE: 93.97 ± 7.03 vs. 55.09 ± 7.08; IL‐1β: 28.02 ± 2.42 vs. 5.50 ± 2.99; IL‐6: 22.95 ± 1.70 vs. 17.41 ± 1.00; COX‐2: 0.582 ± 0.039 vs. 0.360 ± 0.044; *p* < 0.001 for all comparisons).

Treatment with a JAK inhibitor significantly reduced the expression of these markers compared to the D‐gal group (AGE: 61.43 ± 6.63 vs. 93.97 ± 7.03, *p* < 0.001; IL‐1β: 14.98 ± 2.30 vs. 28.02 ± 2.42, *p* < 0.001; IL‐6: 17.49 ± 1.39 vs. 22.95 ± 1.70, *p* < 0.001; COX‐2: 0.428 ± 0.051 vs. 0.582 ± 0.039, *p* < 0.001; Supporting Information 4: Figure S4).

#### 3.5.4. JAK Inhibitors Suppress D‐gal‐Induced ECM Degradation In Vivo

**Figure 7:**
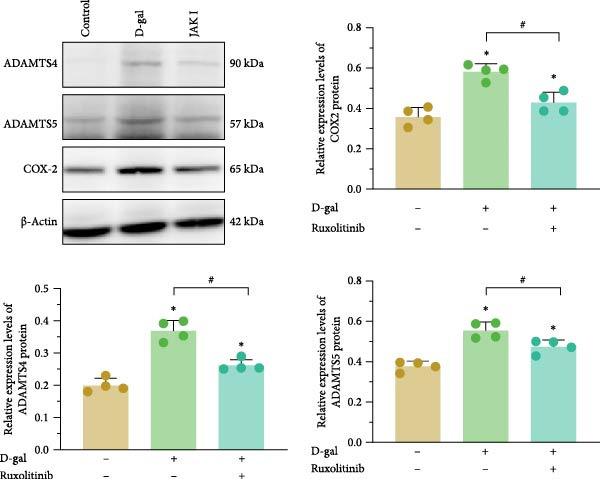
Protein immunoblotting bands and relative expression of ADAMTS‐4, ADAMTS‐5, and COX‐2 in medullary tissues. K–S tests were performed to assess normality of the variables. One‐way ANOVA was used to analyze group differences, with post hoc comparisons conducted via LSD and Tamhane’s tests, and Levene’s test used to assess homogeneity of variance.  ^∗^ denotes a statistically significant difference compared with the control group (*p* < 0.05); # denotes a statistically significant difference between the two groups (*p* < 0.05); *n* = 4.

The expression levels of MMP‐3, MMP‐13, ADAMTS‐4, and ADAMTS‐5 were elevated (0.971 ± 0.023; 425.73 ± 9.08; 0.531 ± 0.083; 0.473 ± 0.068) compared with those in the reference group (0.368 ± 0.059; 266.08 ± 18.26; 0.303 ± 0.039; 0.300 ± 0.020; *p* < 0.001 for all). Concurrently, the expression levels of proteoglycan and Col II were significantly reduced (32.22 ± 2.70; 17.78 ± 3.47 vs. 61.89 ± 1.26; 39.61 ± 2.43; *p* < 0.001 for both; Figure 7).

Following treatment with a JAK inhibitor, the expression levels of MMP‐3, MMP‐13, ADAMTS‐4, and ADAMTS‐5 were reduced (0.560 ± 0.023; 271.55 ± 13.75; 0.397 ± 0.016; 0.319 ± 0.042), compared with the reference values (0.971 ± 0.023; 425.73 ± 9.08; 0.531 ± 0.083; 0.473 ± 0.068; *p* < 0.001, *p* < 0.001, *p* = 0.005, and *p* = 0.001, respectively). Proteoglycan and Col II levels were also significantly increased after JAK inhibitor intervention (Figure 7). These findings suggest that JAK inhibitors attenuate ECM degradation in the setting of D‐gal exposure.

Aggrecan protein was predominantly localized in chondrocytes and the surrounding matrix. In the control group, aggrecan staining was more extensive, with deeper coloration and greater staining intensity compared to the D‐gal‐induced model group. The D‐gal + JAK inhibitor group showed slightly enhanced staining and a higher degree of positive aggrecan expression than the D‐gal model group (Supporting Information 5: Figure S5).

Col II protein was mainly expressed in the cytoplasm of chondrocytes. The control group exhibited a broader distribution and stronger staining intensity of collagen II. In contrast, the D‐gal‐induced group showed lighter staining and a reduced positive area. Treatment with the JAK inhibitor led to a modest increase in staining intensity and distribution of collagen II expression compared to the D‐gal model group (Supporting Information 6: Figure S6).

#### 3.5.5. Histopathological Staining Results

In the control group, the NP of the rat caudal intervertebral disc exhibited a clearly defined morphology, with a normal and evenly distributed population of NPCs. The annulus fibrosus displayed an orderly structure with a uniformly distributed cartilaginous matrix, and the endplates showed no evidence of fractures.

In contrast, the D‐gal‐induced model group demonstrated irregular morphology of the NP, accompanied by a marked reduction in NPCs, which were more sparsely distributed and separated by a larger volume of matrix. The annulus fibrosus exhibited hyperplasia and protrusion toward the NP, along with a reduction in the cartilaginous matrix and visible endplate fractures.

In the D‐gal‐induced model + JAK inhibitor group, the morphology of the NP appeared relatively preserved. A modest increase in NPC number and a reduction in annulus fibrosus hyperplasia were observed. The cartilaginous matrix within the annulus fibrosus showed partial restoration, and the endplates appeared intact (Figure 8).

Figure 8Histological evaluation of IVDD in rat models. HE staining (a), Safranin O/Fast Green staining (b), and corresponding statistical analysis (c). Histological scores were calculated to assess IVDD.  ^∗^ indicates statistically significant differences between the two groups (*p* < 0.05).

**Figure:**
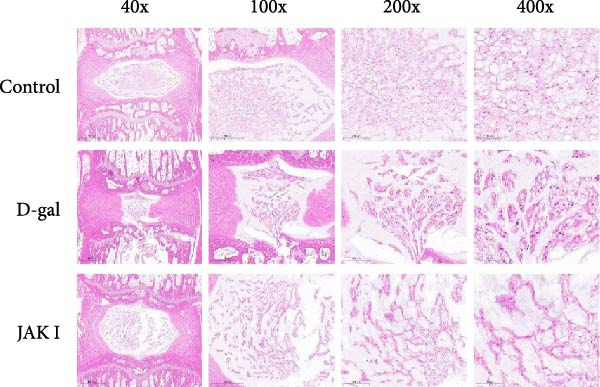
(a)

**Figure:**
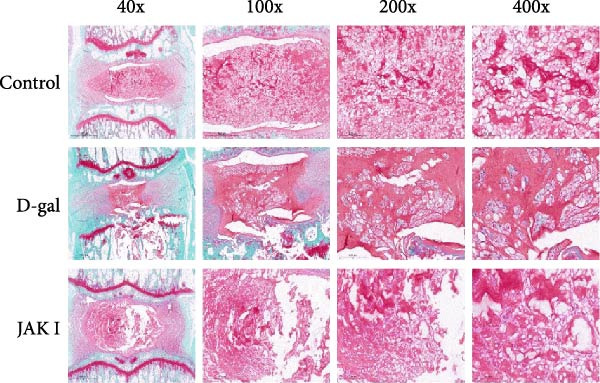
(b)

**Figure:**
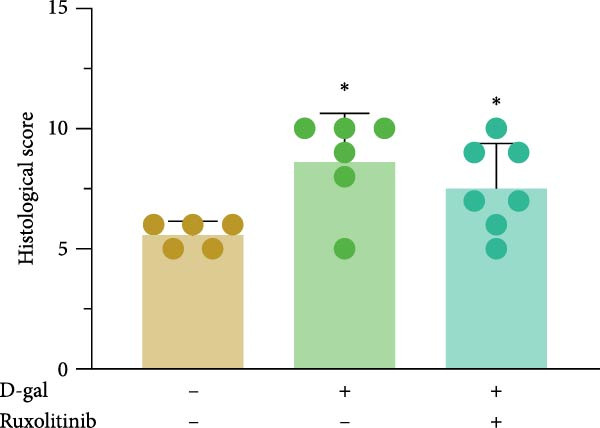
(c)

#### 3.5.6. Expression of JAK2/STAT3 Pathway in Rat Medullary Tissues

**Figure 9:**
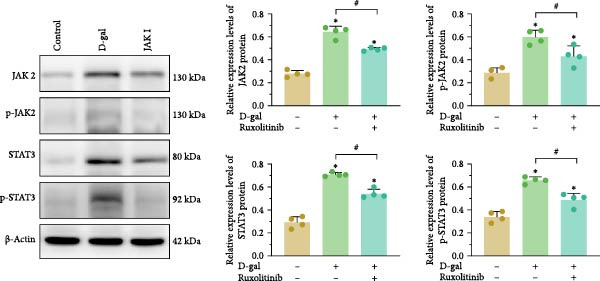
Expression of JAK2/STAT3 pathway proteins in vivo. Normality of phosphorylated protein variables was verified using K‐S tests. One‐way ANOVA was applied to analyze intergroup differences, followed by LSD and Tamhane’s tests for post‐hoc comparisons.  ^∗^ denotes a statistically significant difference compared with the control group (*p* < 0.05); # denotes a statistically significant difference between the two groups (*p* < 0.05).

The expression levels of JAK2, p‐JAK2, STAT3, and p‐STAT3 were analyzed in the medullary tissues of rats across the experimental groups. The results were consistent with those observed in the cellular studies. The D‐gal group demonstrated elevated expression levels of JAK2, p‐JAK2, STAT3, and p‐STAT3, whereas these expression levels were significantly reduced in the JAK inhibitor‐treated group (Figure 9). These findings suggest abnormal activation of the JAK2/STAT3 signaling pathway in intervertebral discs undergoing degeneration.

## 4. Discussion

The pathogenesis of IVDD is multifactorial, involving genetic susceptibility, cellular senescence, oxidative stress, as well as lifestyle, occupational, and environmental factors [10]. This process is characterized by imbalances in ECM synthesis and degradation, apoptosis, inflammation, and abnormal angiogenesis and neurogenesis [11, 12]. In this study, NPCs exposed to D‐gal displayed a senescent phenotype, evidenced by elevated expression of SASP markers (p16, p53, and p21), increased β‐gal staining positivity, and cell cycle arrest.

AGEs are compounds formed via nonenzymatic glycation under hyperglycemic conditions, characterized by brown pigmentation, protein cross‐linking, and protein stabilization [13]. AGE accumulation has been implicated in the progression of IVDD [14]. In this study, AGE expression was significantly higher in the D‐gal‐induced aging group than in control. AGE accumulation induces ECM cross‐linking, increasing disc stiffness and brittleness [15], triggers apoptosis through the activation of apoptotic pathways, decreases cell viability and proliferation, and thus, impairs the reparative capacity of NPCs [16]. AGEs also induce inflammatory responses, enhancing protease activity and contributing to disc degeneration [17].

To determine the effects of D‐gal concentration on the induction of NPC senescence, changes in cellular senescence were assessed using β‐gal staining, western blotting, and ELISA. β‐gal staining showed that treatment with 10 mg/mL D‐gal significantly increased the number of β‐gal‐positive cells. In addition, both protein and RNA expression levels of SA markers—p16, p21, and p53—were significantly elevated. As β‐gal activity and expression of p16, p21, and p53 are key markers of cellular senescence, these findings confirm that 10 mg/mL D‐gal effectively induces senescence in NPCs.

Chronic sterile inflammation is a hallmark of aging, with elevated levels of proinflammatory cytokines strongly associated with age‐related diseases [18]. Inflammatory injury can disrupt the structural integrity of intervertebral discs and promote NPC senescence. Senescent NPCs produce a SASP, which includes proinflammatory cytokines, chemokines, and MMPs. This secretory profile reinforces chronic inflammation and forms a self‐perpetuating feedback loop that accelerates degeneration [19].

In this study, senescent NPCs demonstrated a strong ability to induce SASP, as indicated by significantly elevated expression levels of IL‐1β, IL‐6, TNF‐α, and COX‐2. These results suggest that senescent NPCs may exacerbate IVDD. Intervention with JAK inhibitors or JAK2 silencing led to a reduction in the expression of these proinflammatory markers. Since the JAK/STAT signaling pathway regulates the expression of numerous proinflammatory cytokines, its inhibition represents an effective strategy for suppressing SASP. The findings showed that both JAK2 silencing and JAK inhibitor treatment effectively reduced SASP expression in senescent NPCs, underscoring the pivotal role of the JAK2/STAT3 signaling pathway in SASP regulation and highlighting its therapeutic potential in mitigating disc degeneration.

JAK inhibitor treatment significantly reduced D‐gal‐induced SA‐β‐gal activity and decreased the expression of SA proteins p53, p21, and p16 in senescent NPCs. This intervention alleviated cell cycle arrest and enhanced the synthesis of aggrecan and Type II collagen. In addition, JAK inhibitors downregulated the phosphorylation of JAK2 and STAT3, thereby inhibiting activation of the JAK2/STAT3 pathway. These inhibitors also mitigated D‐gal‐induced reductions in aggrecan and Type II collagen expression and limited ECM degradation. Collectively, these results indicate that JAK inhibitor treatment suppresses the phosphorylation of JAK2 and STAT3, delays medullary cell senescence, and promotes the synthesis of ECM components in rat models.

Apoptosis plays a key role in the progression of IVDD, as excessive apoptosis of NPCs directly reduces cell viability within the intervertebral disc. In this study, treatment with JAK inhibitors significantly reduced D‐gal‐induced apoptosis in senescent NPCs. Following D‐gal stimulation, the expression levels of ADAMTS4, ADAMTS5, MMP‐3, and MMP‐13 increased, while the levels of Col II and aggrecan decreased. This imbalance led to metabolic abnormalities in NPCs, characterized by reduced ECM synthesis and enhanced ECM degradation within the NP.

Protein expression analyses in rat medullary cells and tissues revealed significant upregulation of ADAMTS4, ADAMTS5, MMP‐3, and MMP‐13, along with downregulation of aggrecan and Col II following D‐gal stimulation. Immunohistochemical staining further confirmed that D‐gal reduced aggrecan and Col II expression, key components of the ECM, while increasing the expression of ECM‐degrading enzymes in cultured human medullary tissues. These findings suggest that D‐gal disrupts metabolic homeostasis in NPCs, promoting ECM degradation.

In contrast, treatment with JAK inhibitors mitigated the D‐gal‐induced loss of aggrecan and collagen II, thereby reducing ECM degradation in NPCs during IVDD. In vivo, NP tissue in the control group exhibited abundant ECM, with highly hydrophilic proteoglycans forming a gel‐like structure that produced high signal intensity on T2‐weighted MRI images. In the D‐gal‐induced degeneration model, progressive loss of proteoglycans led to fibrotic changes in the NP and a corresponding decline in MRI signal intensity.

Histological analysis using HE staining showed that JAK inhibitor treatment partially restored the structural abnormalities in intervertebral discs induced by D‐gal. In addition, Flip‐Red‐O‐Solid‐Green staining demonstrated that JAK inhibitors alleviated the extensive proteoglycan loss and disrupted distribution caused by D‐gal exposure. Treatment also led to a reduction in β‐gal activity and decreased expression of aging‐related proteins in the NP, supporting the potential of JAK inhibitors to attenuate D‐gal‐induced IVDD.

This study demonstrates a strong association between activation of the JAK2/STAT3 signaling pathway and NPC senescence. The JAK/STAT pathway acts as a central communication axis in the regulation of diverse cellular processes [20]. JAK proteins, which are noncovalently linked to cytokine receptors, mediate tyrosine phosphorylation events that lead to the recruitment and activation of STAT proteins. Once phosphorylated, STATs dimerize and translocate to the nucleus, where they regulate the transcription of specific target genes. While different cytokines can activate overlapping sets of STATs, individual STAT proteins often exert distinct and non‐redundant biological effects [21].

The clinical success of small‐molecule JAK inhibitors in the treatment of rheumatic diseases demonstrates the feasibility of targeting intracellular signaling pathways for therapeutic purposes. Tofacitinib, the first JAK inhibitor approved by the U.S. Food and Drug Administration (FDA) for rheumatoid arthritis, has also been investigated for use in other autoimmune conditions. Several additional JAK inhibitors are currently undergoing preclinical and clinical evaluation [22, 23].

Previous studies further support the involvement of the JAK2/STAT3 signaling pathway in IVDD. For example, one study demonstrated that leptin promotes catabolic activity in rat NPCs by upregulating the expression of MMP‐1, MMP‐13, ADAMTS4, and ADAMTS5 via activation of the JAK2/STAT3 pathway, suggesting a mechanism contributing to disc degeneration [24]. Another study reported that resveratrol suppressed IL‐6 expression and attenuated STAT3 phosphorylation, thereby improving NPC viability and function and offering a protective effect against degeneration [25]. Together, these findings highlight the therapeutic potential of JAK/STAT pathway inhibition in alleviating cellular senescence.

To assess JAK/STAT pathway activity in senescent NPCs, protein levels of p‐JAK2 and p‐STAT3 were compared between control and D‐gal‐induced groups. Both markers were significantly upregulated in the D‐gal group. To further explore this activation, an IL‐6 treatment group was included as an overexpression model. IL‐6 stimulation induced a senescent phenotype similar to that observed in the D‐gal group, further confirming IL‐6–mediated JAK2/STAT3 activation.

To determine the specific role of the JAK2/STAT3 pathway in NPC senescence, D‐gal‐induced senescent NPCs were treated with either JAK2‐siRNA or a JAK inhibitor. Both interventions significantly reduced the number of β‐gal‐positive cells and lowered the expression of SA proteins, including p21, p16, p53, and p‐p53.

Cell cycle analysis revealed that the accumulation of senescent NPCs in the G1 phase was reversed following JAK2 knockdown or inhibition. These findings suggest that the JAK2/STAT3 signaling pathway plays a key role in regulating NPC senescence. As NPCs progressively lose their self‐renewal capacity during IVDD, the JAK/STAT pathway may also contribute to maintaining this function.

Furthermore, this study demonstrated that inhibition of the JAK2/STAT3 pathway significantly enhanced the proliferation of senescent NPCs, supporting its potential therapeutic role in restoring the self‐renewal capacity of these cells. Taken together, these results suggest that targeting the JAK/STAT signaling pathway may offer a promising strategy for managing IVDD.

JAK inhibitors have demonstrated significant efficacy in the treatment of inflammatory and autoimmune diseases; however, their off‐target effects and modulation of secondary pathways may complicate therapeutic outcomes. Inhibition of JAK2 can impact hematopoiesis, leading to anemia or thrombocytopenia, necessitating regular blood monitoring in clinical settings [26]. The efficacy of JAK inhibitors must be carefully weighed against potential risks, including infection, thrombosis, and malignancy.

Even when the JAK‐STAT pathway is targeted, compensatory activation of other signaling pathways, such as NF‐κB, MAPK, or PI3K‐mTOR, may occur to maintain inflammation or promote cell survival [27–30]. Although rat and human JAK isoforms (JAK1/2/3/TYK2) are highly homologous, differences in STAT‐regulated gene expression may exist between species. Moreover, human NP cells are more sensitive to inflammatory stimuli such as IL‐6 and TNF‐α, which may influence the anti‐inflammatory response to JAK inhibitors.

Most existing studies rely on acute injury models, whereas human IVDD is a chronic and progressive disease. As a result, the long‐term therapeutic efficacy of JAK inhibitors may be overestimated. In addition, human intervertebral discs are avascular, and drug delivery relies primarily on diffusion, which is inefficient. Given their short plasma half‐life, JAK inhibitors require frequent administration, increasing the risk of systemic toxicity. Although local delivery via percutaneous disc injection is feasible, repeated punctures may accelerate degeneration.

In addition to the aforementioned challenges in clinical translation, this study has several limitations that warrant consideration. First, although ruxolitinib is approved by the U.S. FDA for the treatment of myelofibrosis—supporting its relative safety—this study did not investigate its adverse effects or conduct in vivo safety assessments in rats, such as blood cell counts or organ toxicity evaluations. These data are essential for supporting further clinical development and thus represent a notable gap that will be addressed in future experiments.

Second, although the study demonstrated that the JAK inhibitor reduced JAK2 and STAT3 phosphorylation, thereby inhibiting the JAK2/STAT3 pathway and lowering SASP expression, its precise mechanism of action requires further clarification. Specifically, the inhibitor’s effect on JAK1, its regulation of downstream signaling, and its direct influence on NPC senescence remain unclear and warrant further experimental validation.

These limitations underscore the need for more comprehensive investigations to fully elucidate the therapeutic potential and mechanisms of JAK inhibitors in the context of IVDD.

## 5. Conclusions

JAK inhibitors suppress D‐gal‐induced production of inflammatory factors and oxidative stress markers in NPCs, inhibit ECM degradation and NPC apoptosis, and reduce cellular senescence by modulating the JAK2/STAT3 signaling pathway. These effects contributed to the alleviation of IVDD in rats. While IVDD involves a complex interplay of mechanisms and signaling pathways, this study specifically focused on NPC apoptosis, inflammatory factor production, oxidative stress, ECM degradation, and the JAK2/STAT3 axis. Further research is needed to explore additional regulatory mechanisms contributing to IVDD in rat models.

To enhance therapeutic efficacy, sustained‐release systems such as nanoparticles and hydrogels should be developed to improve drug retention and targeted delivery. The translation of JAK inhibitors into human IVDD therapy faces significant challenges, including species‐specific differences, drug delivery barriers, long‐term safety concerns, and clinical feasibility. Although promising results were observed in rats, future studies should employ models that more closely resemble human physiology and incorporate innovative delivery strategies along with rigorously designed clinical trials to support potential clinical applications.

**Nomenclature**
ACAN: Aggrecan
ADAMTS: A disintegrin and metalloproteinase with thrombospondin motifs
BSA: Bovine serum albumin
CCK‐8: Cell counting kit‐8
DAPI: 4^′^,6‐Diamidino‐2‐phenylindole
ECM: Extracellular matrix
FBS: Fetal bovine serum
IVD: Intervertebral disc
IVDD: Intervertebral disc degeneration
IL‐1β: Interleukin‐1β
IL‐6: Interleukin‐6
JAKs: Janus‐associated kinases
MAPK: Mitogen‐activated protein kinase
MMP: Matrix metalloproteinase
mTOR: Mechanistic target of rapamycin
NF‐κB: Nuclear factor‐kappa B
NP/NPCs: Nucleus pulposus/nucleus pulposus cells
PBS: Phosphate‐buffered saline
ROS: Reactive oxygen species
SASP: Senescence‐associated secretory phenotype
SA‐β‐gal: Senescence‐associated β‐galactosidase
STAT: Signal transducer and activator of transcription
WB: Western blot.

## Ethics Statement

This study was conducted with approval from the Experimental Animal Ethics Committee of Xinjiang Medical University (Approval Number: IACUC‐JT‐20230321‐99). All applicable international, national, and/or institutional guidelines for the care and use of animals were followed.

## Consent

The authors have nothing to report.

## Disclosure

All authors have read and approved the final draft.

## Conflicts of Interest

The authors declare no conflicts of interest.

## Author Contributions

Conception, design of the research: Weidong Liang and Shuwen Zhang. Acquisition of data: Xiaoyu Cai, Yao Wang, and Shuwen Zhang. Analysis and interpretation of the data: Xiaoyu Cai, Kup Ya, and Yao Wang. Statistical analysis: Yao Wang, Honggang Hao, and Kup Ya. Obtaining financing: Weidong Liang. Writing of the manuscript: Weidong Liang, Honggang Hao, and Jun Sheng. Critical revision of the manuscript for intellectual content: Weibin Sheng and Jun Sheng.

## Funding

This study was funded by the Regional Projects of National Natural Science Foundation of China (Grant 82160277) and the Natural Science Foundation Youth Fund of Xinjiang Uygur Autonomous Region (Grant 2022D01C825).

## Supporting Information

Additional supporting information can be found online in the Supporting Information section.

## Supporting information


**Supporting Information 1** Figure S1. Quantification of proinflammatory and catabolic markers by ELISA. ELISA was used to measure levels of IL‐1β, IL‐6, TNF‐α, MMP‐3, and MMP‐13 across groups. Variables were first assessed for normality using the K–S test. One‐way ANOVA evaluated intergroup differences, with LSD and Tamhane’s test (for heterogeneous variance) used for post hoc comparisons, depending on variance homogeneity. Mann–Whitney *U* tests were applied for specific pairwise comparisons when normality assumptions were not met.  ^∗^ denotes a statistically significant difference compared with the control group (*p* < 0.05); # denotes a statistically significant difference compared between the two groups (*p* < 0.05); *n* = 3.

**Supporting Information 2** Figure S2. MRI T2‐weighted imaging and Pfirrmann grading of rat intervertebral discs.

**Supporting Information 3** Figure S3. Expression of aging‐related proteins following different treatments. Left: Relative expression levels of aging‐related proteins (p16, p21, p53, and p‐p53) in medullary tissue. Right: Corresponding protein immunoblotting bands. For variables, K–S tests were first conducted to assess normality. One‐way ANOVA was used to evaluate group effects, followed by LSD and Tamhane’s test for post hoc comparisons.  ^∗^ indicates a statistically significant difference compared with the control group (*p* < 0.05); # indicates a statistically significant difference between two groups (*p* < 0.05); *n* = 4.

**Supporting Information 4** Figure S4. ELISA–based quantification of IL‐1β, IL‐6, TNF‐α, MMP‐3, and MMP‐13. Normality was assessed using K–S tests. One‐way ANOVA was conducted to compare groups, followed by LSD (for homogeneous variance) and Tamhane’s test (for heterogeneous variance) for post hoc analysis. Moreover, Mann–Whitney *U* tests were applied for selected pairwise comparisons, without assuming normality.  ^∗^ indicates a statistically significant difference compared with the control group (*p* < 0.05); # indicates a statistically significant difference between two groups (*p* < 0.05); *n* = 6.

**Supporting Information 5** Figure S5. Immunohistochemistry analysis of aggrecan expression in rat intervertebral disc tissues. (A) Representative images of aggrecan staining across groups. (B) Quantitative analysis of aggrecan‐positive staining intensity. D‐gal treatment reduced aggrecan expression compared to the control group, while JAK inhibitor intervention partially restored it.  ^∗^ indicates a statistically significant difference between groups (*p* < 0.05).

**Supporting Information 6** Figure S6. Immunohistochemistry analysis of collagen II expression in rat intervertebral disc tissues. (A) Representative images of collagen II staining across groups. (B) Quantitative analysis of collagen II‐positive staining intensity. D‐gal‐induced degeneration reduced collagen II expression, which was partially reversed by JAK inhibitor treatment.  ^∗^ indicates a statistically significant difference between groups (*p* < 0.05).

## Data Availability

All data generated or analyzed during this study are included in this article. Further enquiries can be directed to the corresponding author.
