# Supplementary figures and images for: Inhibition of the JAK2/STAT3 Pathway Attenuates D‐Galactose‐Induced Nucleus Pulposus Cell Senescence and Intervertebral Disc Degeneration
Source: Stem Cells Int. 2025 Dec 9;2025:3373211. doi: 10.1155/sci/3373211 (PMC12767374; doi:10.1155/sci/3373211)

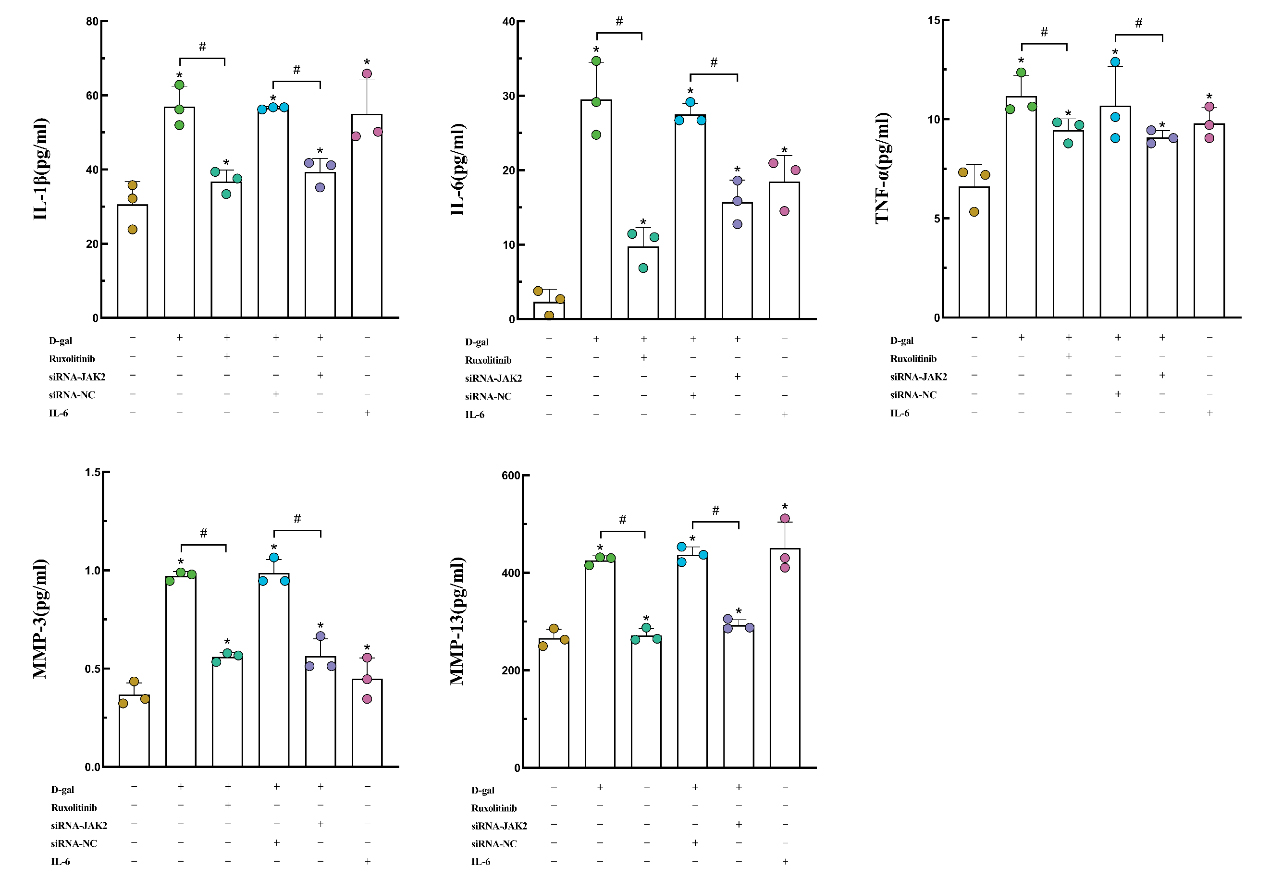

Supplement: Supplementary file 1 — Supporting Information 1 Figure S1. Quantification of proinflammatory and catabolic markers by ELISA. ELISA was used to measure levels of IL‐1β, IL‐6, TNF‐α, MMP‐3, and MMP‐13 across groups. Variables were first assessed for normality using the K–S test. One‐way ANOVA evaluated intergroup differences, with LSD and Tamhane’s test (for heterogeneous variance) used for post hoc comparisons, depending on variance homogeneity. Mann–Whitney U tests were applied for specific pairwise comparisons when normality assumptions were not met. ∗ denotes a statistically significant difference compared with the control group (p < 0.05); # denotes a statistically significant difference compared between the two groups (p < 0.05); n = 3. [file SCI-2025-3373211-s002.jpg]

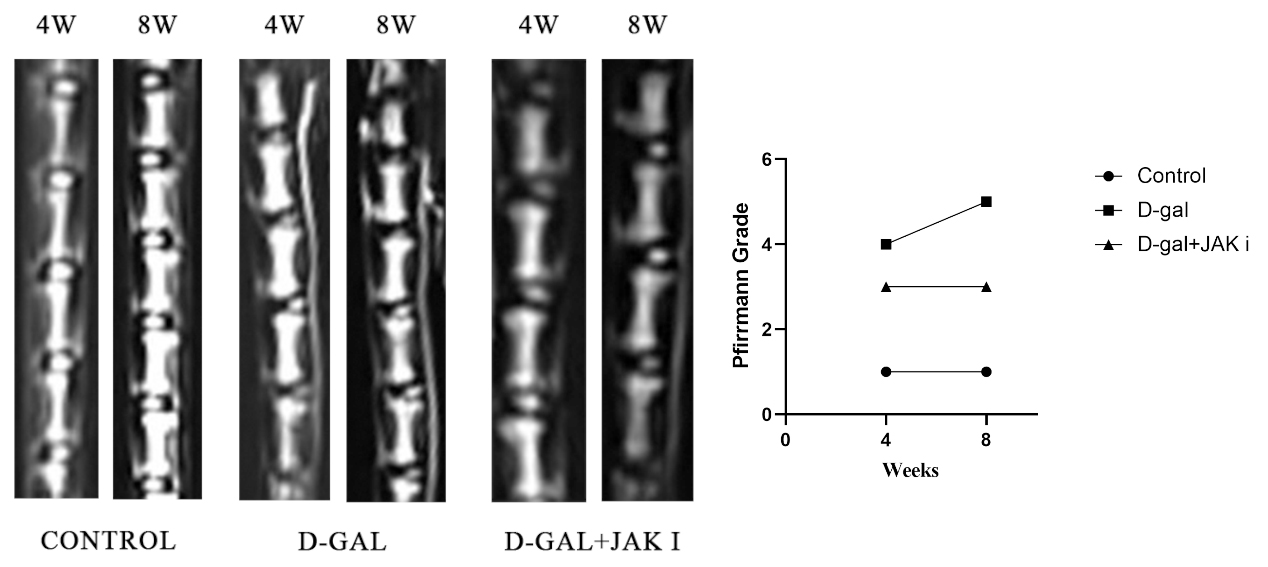

Supplement: Supplementary file 2 — Supporting Information 2 Figure S2. MRI T2‐weighted imaging and Pfirrmann grading of rat intervertebral discs. [file SCI-2025-3373211-s004.jpg]

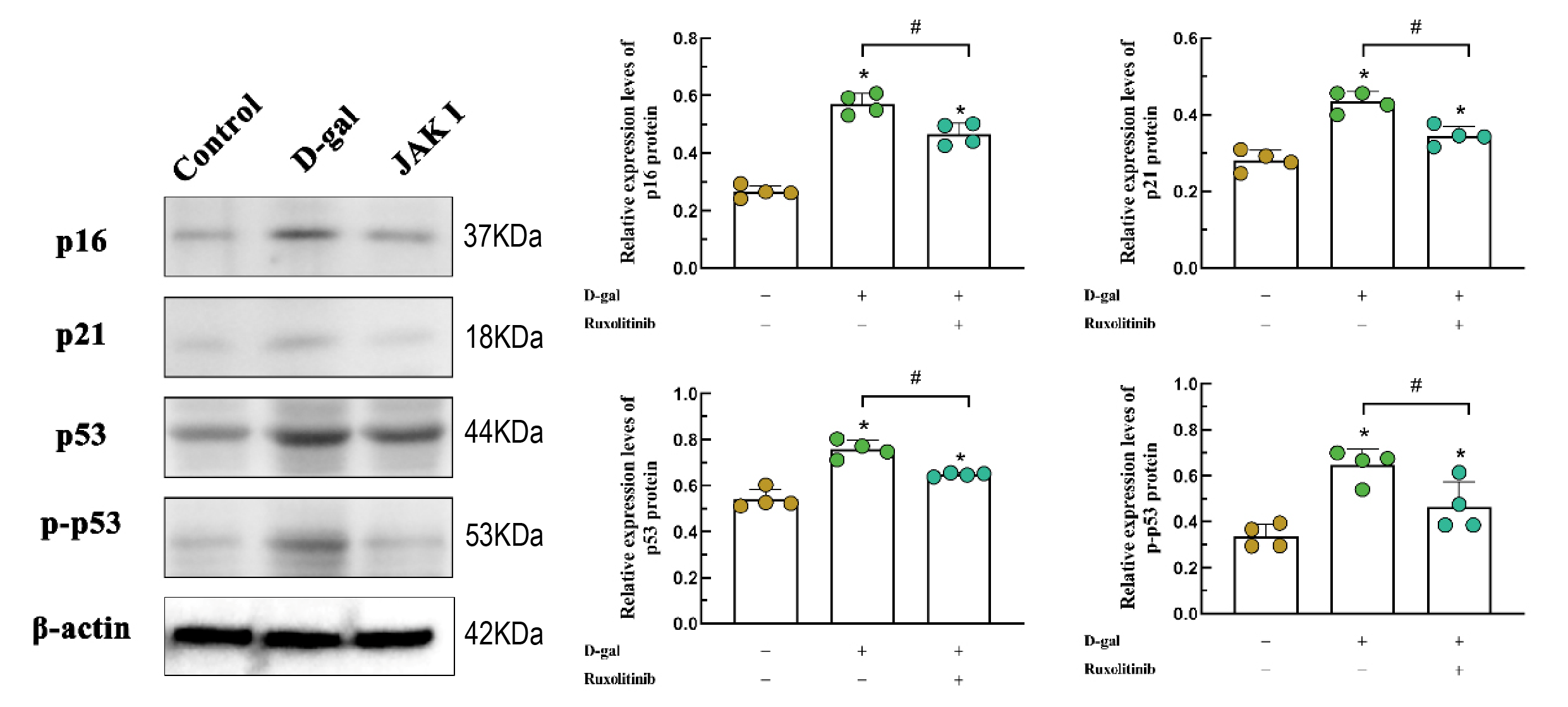

Supplement: Supplementary file 3 — Supporting Information 3 Figure S3. Expression of aging‐related proteins following different treatments. Left: Relative expression levels of aging‐related proteins (p16, p21, p53, and p‐p53) in medullary tissue. Right: Corresponding protein immunoblotting bands. For variables, K–S tests were first conducted to assess normality. One‐way ANOVA was used to evaluate group effects, followed by LSD and Tamhane’s test for post hoc comparisons. ∗ indicates a statistically significant difference compared with the control group (p < 0.05); # indicates a statistically significant difference between two groups (p < 0.05); n = 4. [file SCI-2025-3373211-s003.tif]

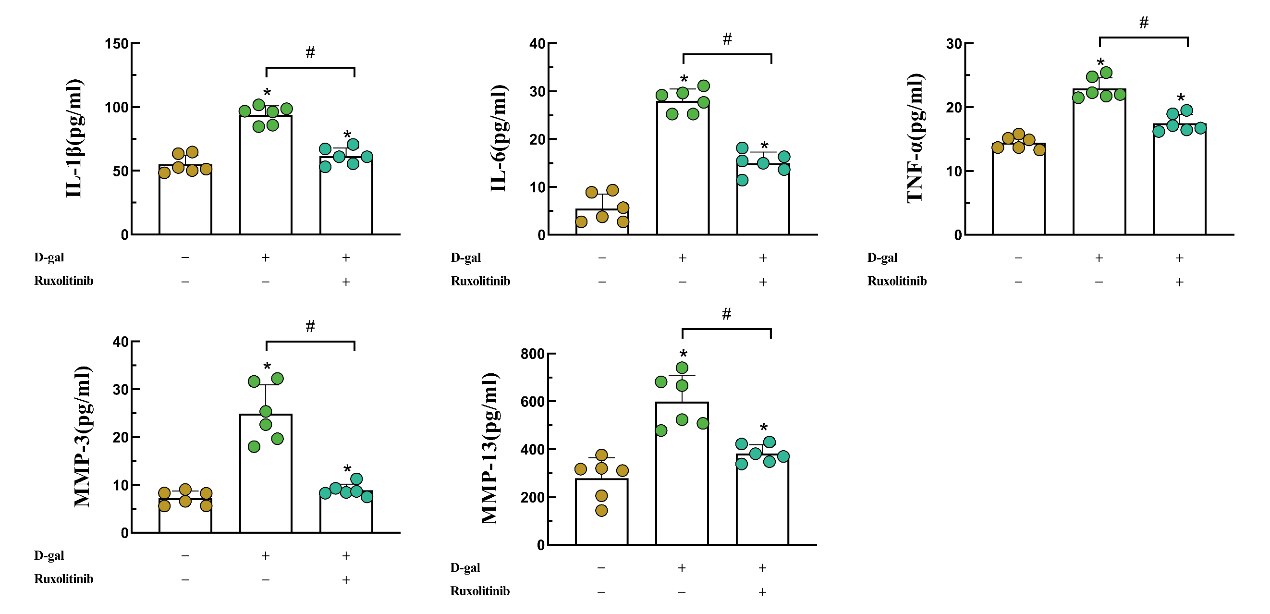

Supplement: Supplementary file 4 — Supporting Information 4 Figure S4. ELISA–based quantification of IL‐1β, IL‐6, TNF‐α, MMP‐3, and MMP‐13. Normality was assessed using K–S tests. One‐way ANOVA was conducted to compare groups, followed by LSD (for homogeneous variance) and Tamhane’s test (for heterogeneous variance) for post hoc analysis. Moreover, Mann–Whitney U tests were applied for selected pairwise comparisons, without assuming normality. ∗ indicates a statistically significant difference compared with the control group (p < 0.05); # indicates a statistically significant difference between two groups (p < 0.05); n = 6. [file SCI-2025-3373211-s006.jpg]

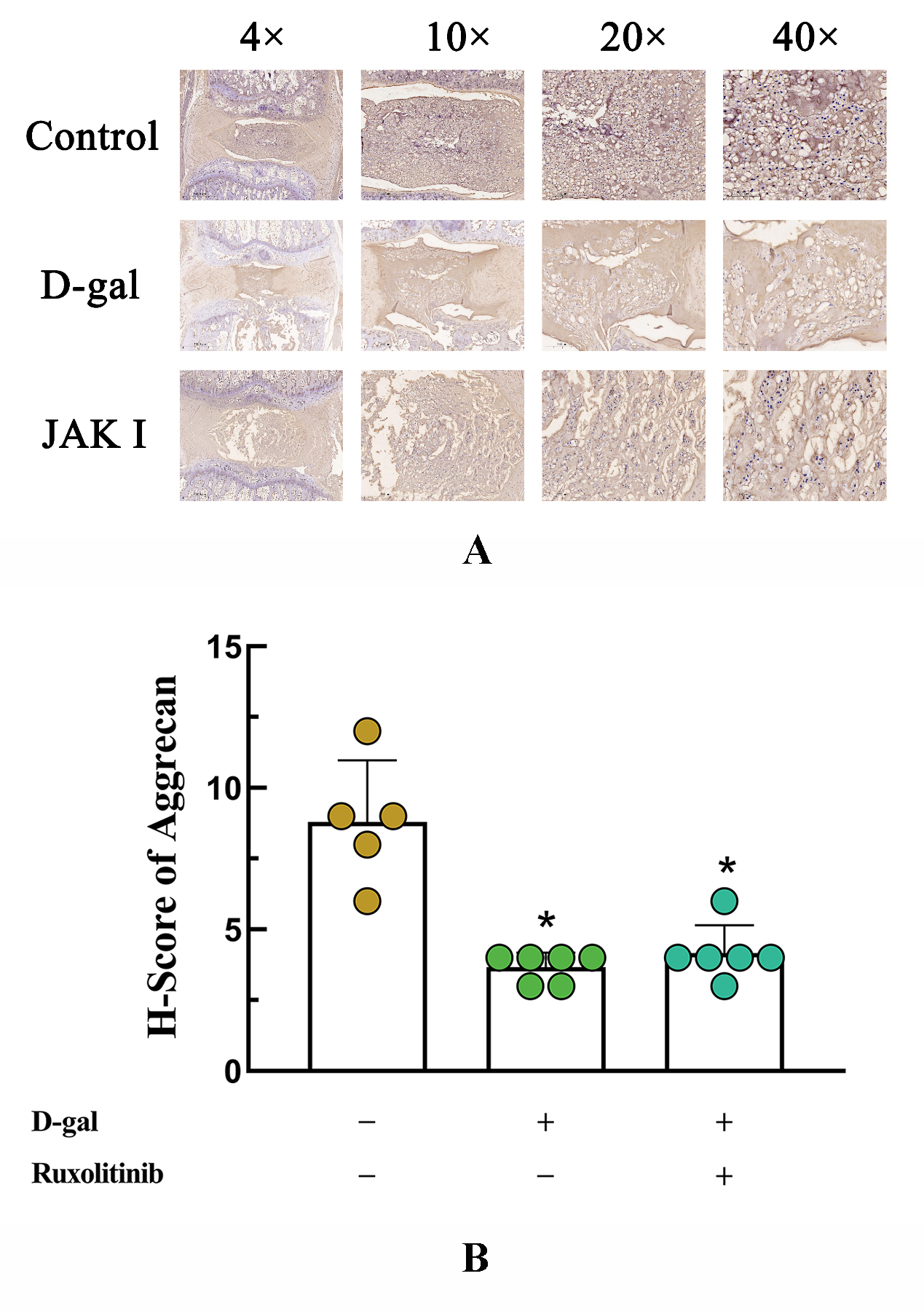

Supplement: Supplementary file 5 — Supporting Information 5 Figure S5. Immunohistochemistry analysis of aggrecan expression in rat intervertebral disc tissues. (A) Representative images of aggrecan staining across groups. (B) Quantitative analysis of aggrecan‐positive staining intensity. D‐gal treatment reduced aggrecan expression compared to the control group, while JAK inhibitor intervention partially restored it. ∗ indicates a statistically significant difference between groups (p < 0.05). [file SCI-2025-3373211-s001.jpg]

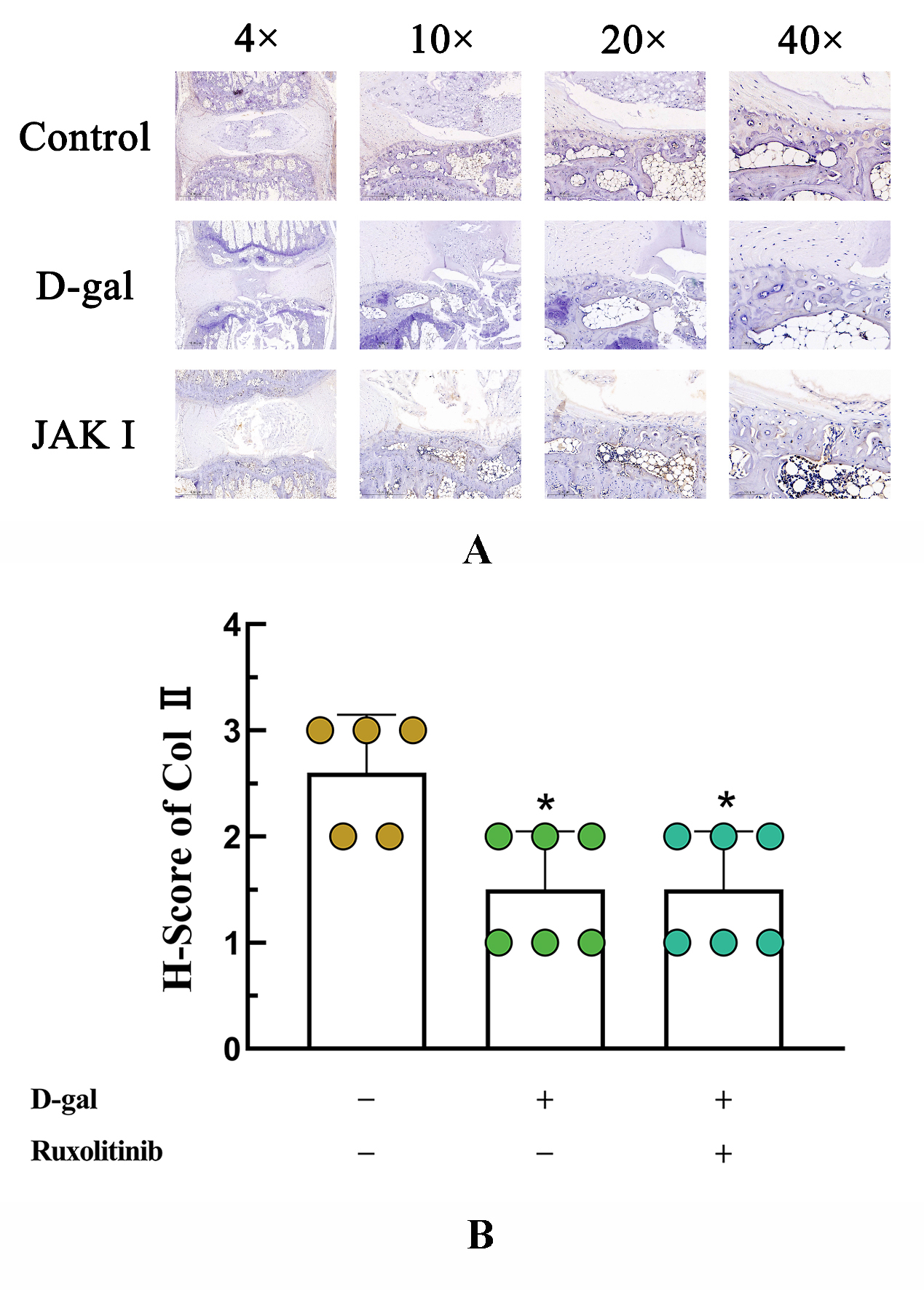

Supplement: Supplementary file 6 — Supporting Information 6 Figure S6. Immunohistochemistry analysis of collagen II expression in rat intervertebral disc tissues. (A) Representative images of collagen II staining across groups. (B) Quantitative analysis of collagen II‐positive staining intensity. D‐gal‐induced degeneration reduced collagen II expression, which was partially reversed by JAK inhibitor treatment. ∗ indicates a statistically significant difference between groups (p < 0.05). [file SCI-2025-3373211-s005.jpg]
